# Enhanced Radiation-tolerant Oxide Dispersion Strengthened Steel and its Microstructure Evolution under Helium-implantation and Heavy-ion Irradiation

**DOI:** 10.1038/srep40343

**Published:** 2017-01-12

**Authors:** Chenyang Lu, Zheng Lu, Xu Wang, Rui Xie, Zhengyuan Li, Michael Higgins, Chunming Liu, Fei Gao, Lumin Wang

**Affiliations:** 1 Key Laboratory for Anisotropy and Texture of Materials (Ministry of Education), School of Materials Science and Engineering, Northeastern University, Shenyang 118109, Liaoning, China; 2 Department of Nuclear Engineering and Radiological Science, University of Michigan, Ann Arbor, MI 48109, United States; 3 China Nuclear Power Technology Research Institute, China General Nuclear Power Corporation, Shenzhen, Guangdong 518000, China; 4 Department of Materials Science and Engineering, University of Michigan, Ann Arbor, MI 48109, United States

## Abstract

The world eagerly needs cleanly-generated electricity in the future. Fusion reactor is one of the most ideal energy resources to defeat the environmental degradation caused by the consumption of traditional fossil energy. To meet the design requirements of fusion reactor, the development of the structural materials which can sustain the elevated temperature, high helium concentration and extreme radiation environments is the biggest challenge for the entire material society. Oxide dispersion strengthened steel is one of the most popular candidate materials for the first wall/blanket applications in fusion reactor. In this paper, we evaluate the radiation tolerance of a 9Cr ODS steel developed in China. Compared with Ferritic/Martensitic steel, this ODS steel demonstrated a significantly higher swelling resistance under ion irradiation at 460 °C to 188 displacements per atom. The role of oxides and grain boundaries on void swelling has been explored. The results indicated that the distribution of higher density and finer size of nano oxides will lead a better swelling resistance for ODS alloy. The original pyrochlore-structured Y_2_Ti_2_O_7_ particles dissolved gradually while fine Y-Ti-O nano clusters reprecipitated in the matrix during irradiation. The enhanced radiation tolerance is attributed to the reduced oxide size and the increased oxide density.

The development of future fusion reactors calls for the materials that can withstand the extreme radiation environments^1^. The first wall/blanket in fusion reactor will be directly bombarded by 14 MeV neutrons to hundreds of displacements-per-atom (dpa) at high temperature, along with large amounts of helium atoms produced by (*n, α*) transmutation reactions^2^. High-density irradiation-induced defect clusters, including dislocation loops, voids and bubbles, may significantly degrade mechanical properties of reactor materials with irradiation hardening, embrittlement and irradiation creep^3^. A category of reduced activation ferritic/martensitic (F/M) steels, nanostructured oxide dispersion strengthened (ODS) steels are considered as promising candidate materials for first wall/blanket applications in fusion reactor because of their promising swelling resistance under irradiation and excellent high-temperature mechanical properties^2^,^4^. Atom probe tomography (APT) and transmission electron microscopy (TEM) revealed that ultra-high density nano-oxides were dispersed homogenously in the steel matrix and at the grain boundaries^2^,^4^,^5^,^6^,^7^. In theory, the interface between matrix and nano-oxide particles can act as efficient trapping sites for point defects and helium atoms to enhance defect recombination and disperse helium atoms, thus minimizing helium embrittlement and suppressing void swelling^4^.

Edmondson *et al*. indicated that nano-scale oxides could disperse helium atoms to promote formation of high-density and ultra-fine helium bubbles in He-implanted 14YWT^8^,^9^. Further, Lu *et al*. found that higher density and finer nano-oxide particles are able to better suppress bubble formation in He-implanted 14Cr-ODS steels^6^. The remarkable irradiation damage tolerance of ODS steels has also been extensively discussed in many previous publications by comparing with non-ODS steels^2^,^4^. However, there’s still a big gap between the microstructures and properties needs to be understood, for example, how the natures and distributions of nano oxides affect the radiation response in ODS steels, and what kind of oxide behaviors will enhance the swelling resistance. In this study, a 9Cr-ODS steel with pre-implanted helium was irradiated by high dose self-heavy ions at evaluated temperature. Microstructural characterizations, including distribution of voids and oxide particles in the ODS steel before and after heavy ion irradiation, were performed by various TEM techniques. A 9Cr-F/M steel with pre-implanted helium was also irradiated in the same condition as a benchmark steel. The swelling results of the 9Cr-F/M steel have been published in another paper^10^, and the swelling value will be compared in this study. The objective of this study is to further understand the relationship between swelling resistance and oxide stability in the irradiated ODS steel.

## Results

### General Microstructure of China ODS steel (COS-2)

**Figure 1:**
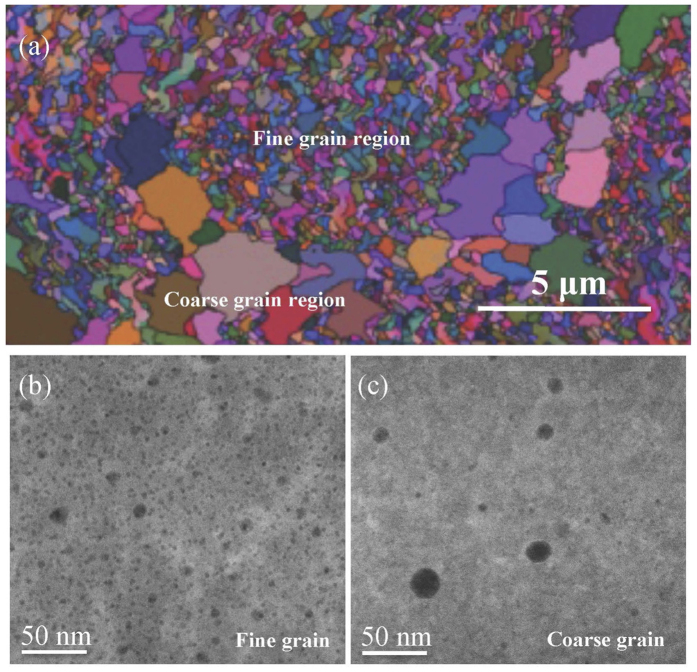
Microstructure of COS-2 before irradiation. (**a**) EBSD mapping showing a bimodal grain distribution, (**b**) HAADF image of nano-oxides in fine grains, (**c**) HAADF image of nano-oxides in coarse grains.

COS-2 is one of the 9Cr-ODS steels developed at Northeastern University in China with a nominal composition of Fe–9Cr–1.5W–0.4Mn–0.1Ta–0.2V–0.3Ti–0.3Y_2_O_3_ (mass%). COS-2 was prepared by mechanical alloying (MA) and spark plasma sintering (SPS). General microstructure of COS-2 characterized by Electron Backscatter Diffraction (EBSD) consists of a ferritic structure with a bimodal grain size distribution as shown in Fig. 1a. The coarse grains are normally larger than 1 μm, while the fine grains are normally smaller than 200 nm. The inhomogeneous temperature gradient during SPS contributes to this special bimodal grain distribution. High angle annular dark field (HAADF) micrographs of coarse grains and fine grains in the unirradiated COS-2 are shown in Fig. 1b and Fig. 1c, respectively. Oxides are shown as dark dots in HAADF images because of their lower mass compared to that of ferritic matrix. It is clear that high-density nano-clusters (NC) distribute homogenously in the fine grains, while larger oxides distribute sparsely in the coarse grains. The average diameter <d> of nano-clusters in fine grains is only 5.9 nm with a number density <N> of 1.8 × 10^22^ /m^3^. In coarse grains, the average size <d> of oxides is 15 nm, and oxides distribute with a much lower density <N> of 1.3 × 10^21^ /m^3^. Bimodal grain distribution has also been observed in a hot-extruded 12Cr ODS alloy reported by Ukai and Chen^11^,^12^. However, the distribution of oxide particles in the fine and coarse grains in 12Cr ODS alloys is exactly contrary to the results in this study. Higher density and finer size of oxide particles were observed in coarse grains, while lower density and larger size of oxide particles were observed in fine grains in hot-extruded 12Cr ODS. This contradiction is because of the dual-phase formation in 12Cr ODS. In 12Cr ODS alloy, ferrite grains (up to ~1 μm) were significantly larger than tempered martensite grains (~200 nm). Therefore, the mechanisms of oxide particle formation in dual-phase are quite different. More details can be found in the reference^11^. However, in this study, COS-2 is a fully ferrite alloy. The bimodal grain distribution is caused by the inhomogeneous temperature gradients during SPS. Temperature variation leads the grains partial recrystallized through dislocation and point defect recovery. The discrepancy of oxide distribution in fine grains and coarse grains is attributed to the Ostwald ripening mechanism^13^. A similar heterogeneous structure in SPSed ODS steel was reported by Boulnat^14^. In the recrystallized grains, high local temperatures may suppress the formation of metastable non-stoichiometric Y-Ti-O nano clusters and accelerate the transformation of NCs into stoichiometric Y_2_TiO_5_ or Y_2_Ti_2_O_7_ oxides, thus decreasing the density and increasing the average size of the oxides in the coarse grains.

### Void swelling

**Figure 2:**
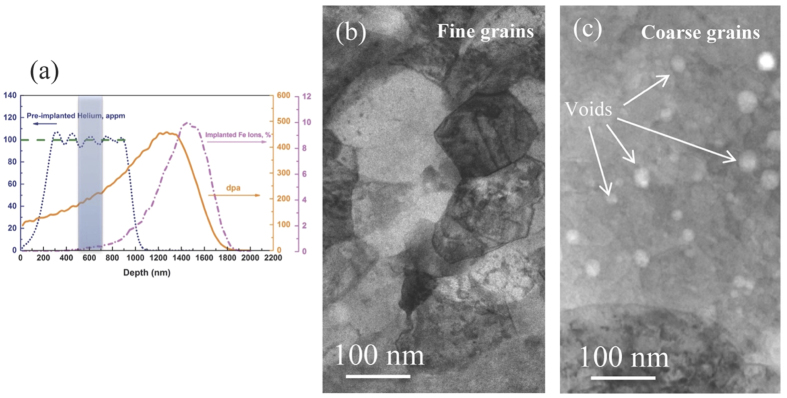
Void swelling in COS-2. (**a**) SRIM prediction profiles of pre-implanted helium distribution, irradiated Fe^++^ ion concentration and displacement damage dose. (**b**~**c**) BF TEM images of COS-2 (b) in fine grain region and (**c**) in coarse grain region irradiated to 188 dpa by 5 MeV Fe^++^ at 460 °C with 100 appm helium concentration.

The Stopping and Range of Ions in Matter 08 (SRIM08) code in Kinchin-Pease mode was used to predict the local displacement damage level, the concentrations of injected Fe ions and the pre-implanted He ions in COS-2^15^. SRIM was calculated with a displacement threshold energy of 40 eV. The predicated profiles are shown in Fig. 2a. In order to avoid the influence of injected interstitial self-ions, the depth range of 450 nm ~750 nm from the surface with a dose of 188 dpa was used for analyzing void swelling in this study. Helium ions were pre-implanted with the following energies, 80, 140, 220, 310, and 420 keV, respectively, to obtain a relatively uniform concentration through the depth range of 300~1000 nm from the sample surface^10^. Bright-field (BF) TEM images of typical area in fine grains and coarse grains of COS-2 after irradiation are shown in Fig. 2b and c. No detectable voids observed in the fine grains, while voids ranging in size from ~5 to 20 nm were found in the coarse grains. The voids are distributed inhomogeneously. Denuded zones appear along the grain boundaries in the coarse grains. The void distribution was evaluated from more than 100 fine grains and 30 coarse grains for the statistical reliability. The details of calculation method on swelling value can be found in another published paper^10^. Compared to non-ODS 9Cr-F/M steel, the ODS-9Cr F/M steel shows significant improvements on swelling resistance. The 9Cr-F/M steel shows a swelling value of ~4.14% after irradiation^10^, much higher than that in the 9Cr ODS steel. As a comparison, the swelling values in fine grains and coarse grains of the 9Cr ODS steel are only 0.05% and 0.36%, respectively.

### Nano-oxide stabilization after heavy ion irradiation

**Figure 3:**
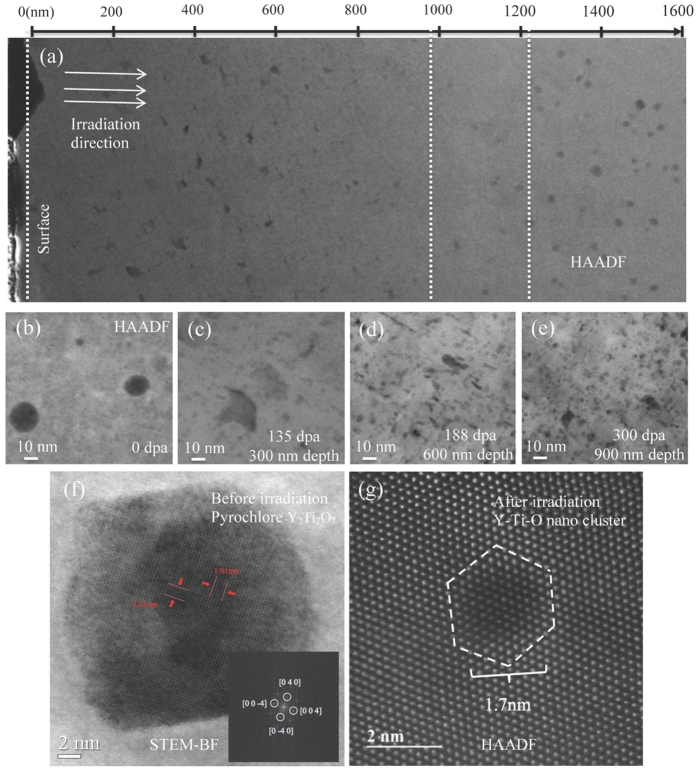
Microstructure changes of COS-2 after heavy ion irradiation. (**a**) Cross-sectional HAADF STEM image of a coarse grain in COS-2 after 5 MeV Fe^++^ irradiation to a fluence of 4.6 × 10^17^ ion/cm^2^ at 460 °C, with 100 appm helium pre-implanted; (**b**) HAADF image showing oxide distribution in coarse grain of COS-2 before irradiation; (**c**)~(**e**) HAADF images showing oxide distributions in various depth with various doses; (**f**) High resolution STEM-BF image showing the pyrochlore-structured Y_2_Ti_2_O_7_ found in the coarse grains before irradiation; (**g**) HR-HAADF image showing the newly formed Y-Ti-O nano-cluster in coarse grains after irradiation.

Many studies show that nano-oxides exhibited high thermal stability^16^,^17^, however, their stabilities under neutron/ion irradiation are still in a debate^18^. In fine grains of COS-2, the density and size distribution of oxide particles show no apparent changes after helium implantation and heavy ion irradiation. Figure 3a shows the cross-sectional HAADF image of the coarse grains in COS-2 after helium implantation and heavy ion irradiation. The incident Fe ions were injected from left to right. Before irradiation, large oxides (normally larger than 10 nm) are spherical and distributed sparsely in the coarse grains as shown in Fig. 3b. However, the shape and distribution of oxide particles change dramatically during irradiation, as shown in Fig. 3a. These changes are depth dependent. From the front surface to a depth of ~950 nm, large oxide particles dissolve gradually and their sizes decrease with increasing depth, and the shape of oxide particles changes from spherical to irregular. In a depth range of 950–1150 nm, the density of large oxide particles is very low. However, in the region deeper than 1200 nm, the distribution of large oxide particles is similar to that in the un-irradiated sample. It is well known that ion irradiation leads to two varied parameters along the depth: displacement damage dose and implanted ion concentration^3^. As shown in Fig. 2a, the highest implanted ion concentration of Fe is at a depth of 1600 nm. No observed changes in the morphology and distribution of oxides are found at this depth. The most significant changes occur near the region with the highest displacement damage dose (1200 nm depth, 450 dpa). Therefore, the modification in oxide distribution should mainly be attributed to cascade collision, but not the variation of the local solubility caused by the implanted Fe ions.

**Figure 4:**
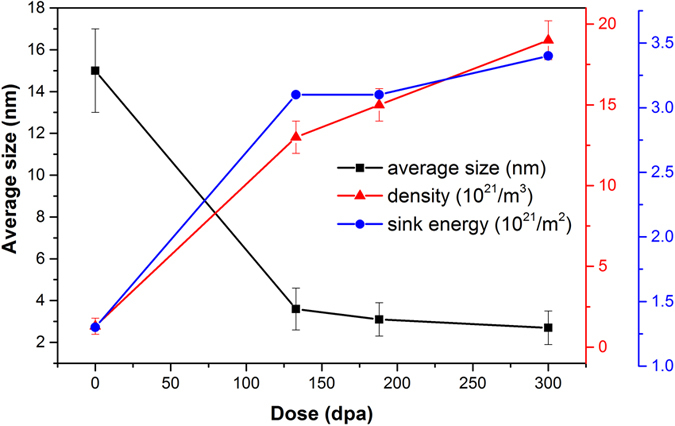
Dependence of oxide size, density and sink energy (S_o_) on increasing ion dose in coarse grains of COS-2.

Typical HAADF images of coarse grains in COS-2 as a function of radiation dose are shown in Fig. 3(b–e), where the representative oxides are shown from the regions irradiated to doses of 0, 135 (300 nm depth), 188 (500 nm), and 300 (900 nm) dpa in the irradiated sample, respectively. Clearly, with increasing the damage level, large oxides dissolve gradually from spherical (0 dpa) to irregular shape (135 dpa), sequentially to needle rod (188 dpa), and then much smaller particles (300 dpa). Meanwhile, a high-density of small oxides is formed after irradiation. The dependence of oxide size and density with increasing ion dose is shown in Fig. 4. It is concluded that the average size of the oxides decreases dramatically and the density increases sharply with increasing damage level.

Many scanning TEM (STEM) images were taken to characterize the structures of the oxides before and after irradiation. Figure 3f shows a typical BF HR-STEM image of a large oxide in a coarse grain before irradiation. The diameter of this oxide is about 10 nm. The Fast Fourier Transform (FFT) analysis of the oxide is embedded in the bottom right and shown a fcc cubic structure observed along the [100] axis. The inter-planer spacing and angles are reasonably consistent with the pyrochlore-structured Y_2_Ti_2_O_7_ with a lattice parameter of *a* = 1.01Ǻ. The corresponding atomic planes of the oxide are (040) and (004). However, after irradiation, the structure of irregular oxides – which are presumed the pre-existing Y_2_Ti_2_O_7_, are difficult to characterize, because of the distorted lattice caused by heavy ion bombardment. The newly formed nano clusters are also characterized, but they no longer present the pyrochlore structure. HR-HAADF image of a typical NC found in a coarse grain after irradiation is shown in Fig. 3g with the electron incidence parallel to the bcc [111]_matrix_ direction. The diameter of this NC is only 1.7 nm; the NC displays faceted interfaces along low-index planes of the matrix. The shape of this NC is shown in a hexagonal shape with facets defined by the {110}_matrix_. NCs have been reported in many previous studies^2^,^4^. Atom probe tomography (APT) studies showed that they were highly nonstoichiometric with high Ti/Y and low O/(Ti + Y) ratios^2^,^4^. The structures of NCs are still under considerable debate. In this study, no distinct lattice structure has been detected in the NCs. This suggests that they are not Y_2_Ti_2_O_7_, Y_2_TiO_5_ or Y_2_O_3_. Hirata *et al*. applied HR-HAADF to the study of NCs and claimed that NCs had a NaCl rock salt structure, which were coherent with the ferritic matrix^5^. Brandes found a similar faceted NC in 14YWT and stated that NCs were amorphous^19^. Higgins conducted molecular dynamic simulations and claimed that NCs below 2 nm were completely disordered^20^. Other studies suggested NCs were non-equilibrium phases or a Guinier-Preston-zone transition phase with solute atoms occupying or nearing the lattice sites of the bcc Fe matrix^2^.

## Discussion

### Mechanisms for enhanced swelling resistance of COS-2

Normally, grain boundaries, dislocations, and the interfaces between the matrix and precipitates are thought to be the preferential sinks for absorbing vacancies and interstitials^1^,^4^,^21^. In this study, the improved swelling resistance of COS-2 is attributed to high-density nano-oxides. When helium is presence, the interfaces between nano-oxides and the matrix can effectively trap helium atoms and disperse helium to prevent coarse bubble formation. The presence of high-pressure gas bubbles further improves irradiation resistance because the ultra-fine bubbles can act as stable residual vacancy-interstitial recombination centers. Actually ultra-fine bubbles are much more effective in enhancing defect recombination than the oxides^4^. On the other hand, the higher density of nano-oxides and grain boundaries in the fine grain region can account for the superior swelling resistance compared to the coarse region^22^,^23^. The high density of interfaces and boundaries in fine grains could support a higher density of trap sites for the recombination of defects. As shown in Fig. 2, the void-denuded zones at grain boundaries confirmed that the swelling suppression due to the stable sinks. The sink strength (number of defect sinks per unit area) of the oxides and the grain boundaries for both regions can be calculated using the following equations^21^,^24^:


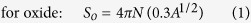



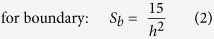


where *S*_*o*_ is the sink strength contributed from oxides, *N* is the number density of the oxides, *A* is the surface area of the oxides, *A* = 4*πr*^2^, *r* is the average radius of the oxides, *S*_*b*_ is the sink strength contributed from grain boundaries, and *h* is the average grain size. Based on the statistics of microstructures in fine grains and coarse grains, the *S*_*o*_ is ~7.1 × 10^14^ m^−2^ for fine grains, almost five times higher than *S*_*o*_ for coarse grains, which is ~1.3 × 10^14^ m^−2.^ The *S*_*b*_ for fine grains is 4 × 10^14^ m^−2^, over an order higher than the *S*_*b*_ for coarse grains, which is ~1.5 × 10^13^ m^−2^. These calculations show that both *S*_*o*_ and *S*_*b*_ in fine grains are much higher than in coarse grains, and hereafter fine grains provide a better swelling resistance than coarse grains. In addition, it is believed the oxides dominate the swelling behavior because of their higher sink strength compared to the grain boundaries in this study. It is worth noting that, there are continuous changes of oxide distributions in coarse grains during the irradiation as shown above. Therefore, the variation in sink energy with increasing dose is also calculated and shown in Fig. 4. It is shown that, although the original oxides in the alloy are unstable during irradiation, the increased density of smaller oxide particles actually provides more trapping sites for absorbing helium and recombining radiation-induced point defects, which can also improve radiation tolerance.

### Dissolution-reprecipitation process of nano-oxide during irradiation

**Figure 5:**
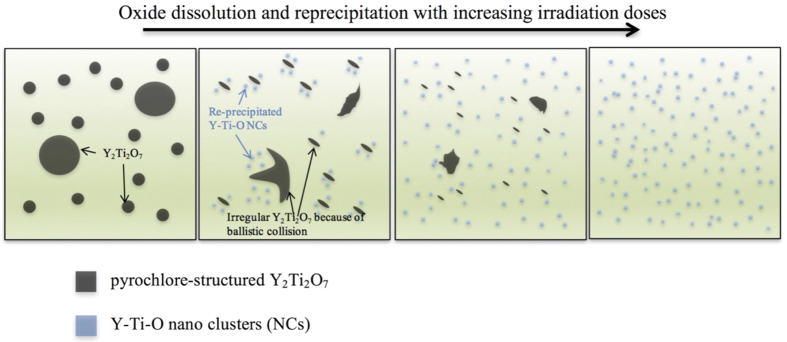
Schematics of oxide dissolution-reprecipitation process in coarse grains of COS-2.

Allen *et al*. systematically summarized the studies on radiation stability of Y-Ti-O oxide^18^. It was found that the stability of oxides depended highly on irradiation conditions, energy, fluence, flux, temperature and the microstructures of ODS alloys. In this work, the oxides are shown to decrease in size and increase in density with the increasing radiation dose. Dissolution of the oxides under high temperature irradiation was also found in other studies. Both Allen^18^ and Monnet^25^ found that the oxides preferred to dissolve at higher temperature and higher doses. Their works indicated that the oxide evolution was not only caused by cascade collision, but also involved the diffusion mechanism. Based on our experimental results, the changes of the oxides can be concluded as dissolution – reprecipitation process. The oxide evolution in coarse grains of COS-2 after irradiation is shown in a schematic in Fig. 5. In the matrix, large size Y_2_Ti_2_O_7_ is distributed sparsely before irradiation. During the irradiation, the solute atoms in oxides are ejected from oxides by high-energy ion bombardment, sequentially diffusing into the ferritic matrix. Once the size of the oxides is similar to the size of the cascades, the oxides are likely to be fully destroyed by cascade collision. However, if the oxide is large, it will be dissolved continuously by increasing radiation fluence, resulting in the observed irregular shapes of Y_2_Ti_2_O_7_. The ballistic dissolution process of the solute atoms can last till the total dissolution of the original oxides. Frost and Russell presented the fate of the dissolved solutes, which depended on the solute diffusivity and solubility^26^. Once the local concentration of solute atoms exceeds the requirements for homogeneous nucleation, NCs/small oxides will reprecipitate from ferritic matrix, but the size of newly formed oxides is smaller than the original oxides. As shown in Fig. 4, a large size Y_2_Ti_2_O_7_ oxides are dissolved, and smaller NCs eventually reprecipitate after irradiation. This explains the changes of the density and size of oxides before and after irradiation in coarse grains of COS-2. The evolution of oxide particles in COS-2 is similar to a previous work conducted by Chen^27^. Chen and his colleagues systematically studied the coherency of the dispersoids before and after irradiation. A model comprehensively established on diffusion recovery, Gibbs-Thomson-effect and radiation damage has been proposed, which can be used to explain the integrated observations in this study. On the other hand, the pre-implanted helium may also have a significant influence on the oxide evolution during irradiation. Helium can facilitate the nucleation of voids by lowering the critical radius required for bias-driven void growth, sequentially increasing the concentration and reducing the size of voids^10^. The newly formed small voids can act as the preferential reprecipitation sites for the dissolved elements of the NCs during the irradiation. Therefore, the dissolution – reprecipitation processing can be enhanced by the existing helium concentration.

In summary, a radiation tolerant 9Cr-ODS steel prepared by MA and SPS containing 100 appm pre-implanted helium was irradiated with 5 MeV Fe^++^ to a fluence of 4.6 × 10^17^ ion/cm^2^ at 460 °C. The microstructures before and after irradiation were characterized by TEM and STEM. The 9Cr ODS steel shows a better swelling resistance than conventional F/M steel due to the existence of high density nano-sized oxides. While pyrochlore-structured Y_2_Ti_2_O_7_ particles became unstable and dissolved gradually under elevated temperature and high dose irradiation, fine Y-Ti-O nano clusters reprecipitated continuously in the matrix. Thus, average oxide size decreased and the oxide density increased with increasing irradiation dose. The increased radiation tolerance of the ODS steel is related to the dissolution–reprecipitation mechanism during high fluence irradiation.

## Methods

COS-2 was prepared by mechanical alloying (MA) and spark plasma sintering (SPS). MA was conducted in a planetary high-energy ball mill (Fritsch P5) at room temperature for 50 h in an argon-gas atmosphere. As-milled powders were consolidated by SPS at 950 °C under a pressure of 40 MPa for 5 min. Mechanically polished COS-2 was pre-implanted with 100 appm helium at room temperature by a 400 keV ion implanter. The ion irradiation was performed using 5 MeV Fe^++^ with a raster beam to a fluence of 4.6 × 10^17^ ions/cm^2^ at a temperature of 460 °C. The dose rate was 3.5 × 10^−4^ dpa/s. The raster beam was used to obtain a uniform irradiation region.

Electron backscatter diffraction (EBSD) was conducted to characterize the morphology and size distribution of the grains in COS-2. TEM samples were prepared using the focused ion beam (FIB) lift-out methods in a FEI Helios Nanolab Dualbeam workstation. Void characterizations were performed on a high resolution TEM JEOL 3011 operated at 300 keV. A double Cs-corrected STEM (JEOL 3100R05) was employed for STEM imaging with an inner angle of 59 mrad and camera length of 15 cm. TEM sample thickness was measured by electron energy loss spectroscopy (EELS)^28^.

## Additional Information

**How to cite this article**: Lu, C. *et al*. Enhanced Radiation-tolerant Oxide Dispersion Strengthened Steel and its Microstructure Evolution under Helium-implantation and Heavy-ion Irradiation. *Sci. Rep.* **7**, 40343; doi: 10.1038/srep40343 (2017).

**Publisher's note:** Springer Nature remains neutral with regard to jurisdictional claims in published maps and institutional affiliations.
